# Recent Insights into the Paradoxical Effect of Echinocandins

**DOI:** 10.3390/jof4010005

**Published:** 2017-12-28

**Authors:** Johannes Wagener, Veronika Loiko

**Affiliations:** 1 Max von Pettenkofer-Institut für Hygiene und Medizinische Mikrobiologie, Medizinische Fakultät, LMU München, 80336 Munich, Germany; loiko@mvp.uni-muenchen.de; 2 Institut für Hygiene und Mikrobiologie, Julius-Maximilians-Universität Würzburg, 97080 Würzburg, Germany

**Keywords:** echinocandin, caspofungin, micafungin, anidulafungin, paradoxical effect, paradoxical growth, glucan synthase, Fks1, antifungals, echinocandins

## Abstract

Echinocandin antifungals represent one of the most important drug classes for the treatment of invasive fungal infections. The mode of action of the echinocandins relies on inhibition of the β-1,3-glucan synthase, an enzyme essentially required for the synthesis of the major fungal cell wall carbohydrate β-1,3-glucan. Depending on the species, echinocandins may exert fungicidal or fungistatic activity. Apparently independent of this differential activity, a surprising in vitro phenomenon called the “paradoxical effect” can be observed. The paradoxical effect is characterized by the ability of certain fungal isolates to reconstitute growth in the presence of higher echinocandin concentrations, while being fully susceptible at lower concentrations. The nature of the paradoxical effect is not fully understood and has been the focus of multiple studies in the last two decades. Here we concisely review the current literature and propose an updated model for the paradoxical effect, taking into account recent advances in the field.

## 1. Introduction

Invasive fungal infections contribute significantly to the morbidity and mortality of immunocompromised patients. Unfortunately, the arsenal of antimycotic drugs is very limited. Current therapy of invasive fungal infections relies on primarily four antifungal drug classes: the polyenes (i.e., amphotericin B), the azoles (e.g., fluconazole, posaconazole, and voriconazole), the pyrimidine analogues (i.e., flucytosin), and the echinocandins (e.g., caspofungin, micafungin, and anidulafungin). While polyenes and azoles directly or indirectly target the fungal membrane and the antimetabolite flucytosin disturbs DNA and RNA synthesis, echinocandins interfere with the fungal cell wall biogenesis by noncompetitively inhibiting the β-1,3-glucan synthase (β-GS, Fks1). This enzyme is responsible for the synthesis of the major fungal cell wall carbohydrate β-1,3-glucan.

The β-GS is a multi-pass membrane protein that is located in the plasma membrane [1]. It is assumed that the enzyme synthesizes a linear β-1,3-glucan polymer using UDP-glucose monomers as a substrate [2,3]. Specialized enzymes may subsequently rearrange the β-1,3-glucan polymers, such as by forming β-1,6-glucan linkages [4], which significantly contribute to the rigidity and dynamics of the cell wall. Depending on the fungal species, approximately one-third to four-fifths of the cell wall consists of β-1,3-glucan and its rearrangement derivatives [5].

Chemical inhibition of the β-GS or deletion of the underlying genes may have fatal consequences for the organism. Yeasts that are lacking the enzymatic activity are typically not viable [6,7,8,9]. Because of this, echinocandins are usually considered fungicidal for susceptible (pathogenic) yeasts. Resistance may occur, but, so far, was always associated with mutation in the β-GS itself [10]. It was proposed that these mutations do not impair the ability of echinocandins to bind to the enzyme but instead reduce the synthesis velocity [10,11,12]. The situation with filamentous fungi in the genus *Aspergillus* is somewhat different. Echinocandins show only a fungistatic activity against these molds [13]. Under in vitro conditions, this is reflected by incomplete growth inhibition, accompanied by significant morphological changes in the mold’s hyphae. The lowest echinocandin concentration that is able to provoke this morphological alteration is therefore called the minimal effective concentration (MEC), which is in contrast to the minimal inhibitory concentration (MIC) usually determined for antimicrobial agents. The reason for the limited activity of echinocandins against *Aspergillus* has not been understood as for a long time β-1,3-glucan was believed to be essential in all fungi. However, we could recently show that the pathogenic mold *Aspergillus fumigatus* is able to survive and grow without β-1,3-glucan. Nevertheless, deletion of the β-GS in *A. fumigatus* has severe consequences for the growth rate, morphotype, and conidiation (sporulation) [14].

In clinical settings, echinocandins show excellent antifungal activity against the most relevant pathogenic *Candida* species and good antifungal activity against most *Aspergillus* species. The frequency of echinocandin resistance is generally low, thereby making echinocandins a first choice for empiric therapy of invasive candidiasis [15,16]. Moreover, echinocandins are recommended for second-line therapy of invasive aspergillosis [16]. Phenotypic antifungal susceptibility testing is routinely performed to avoid potential therapy failure. When performing susceptibility testing under standardized conditions, e.g., with the broth microdilution method, a surprising echinocandin-specific phenomenon called the “paradoxical effect” can be observed. This paradoxical effect is characterized by the ability of certain strains to grow in the presence of higher echinocandin concentrations (approximately 4 to 32 µg mL^−1^) while being fully susceptible at lower concentrations (approximately 0.03 to 1 µg mL^−1^). In the presence of very high concentrations (approximately >64 µg mL^−1^), the fungal species showing the paradoxical effect become susceptible again [17,18,19]. The phenomenon was observed with multiple species in the genera *Candida* and *Aspergillus* [20,21,22]. Within this review we will summarize and discuss the current knowledge and recent advances related to the paradoxical effect of the echinocandins.

## 2. The Paradoxical Effect: Dependence on Species, Strain, and Echinocandin Derivative

A paradoxical effect of an echinocandin was described for the first time in 1988. Hall and colleagues reported for the nonclinical echinocandin cilofungin that many isolates of *Candida albicans* and *Candida tropicalis* continued to grow in concentrations above that of partial inhibition [17]. Later on, this phenomenon was also observed for other echinocandins and further fungal species, including molds of the genus *Aspergillus* [20,21,22]. Interestingly, not every echinocandin derivative seems to have the same capability to evoke the paradoxical effect. Of the three derivatives in clinical use, namely caspofungin, micafungin, and anidulafungin, caspofungin was most frequently associated with paradoxical growth in *Candida* spp. [18,21,22,23,24,25]. For example, of approximately 120 clinical *Candida dubliniensis* isolates that were tested by Fleischhacker et al., 90% showed paradoxical growth with caspofungin, 63% with micafungin, but none with anidulafungin [22]. Similar observations were made with different isolates of *Aspergillus* spp. [20,26]. Of 11 clinical isolates six showed paradoxical growth with caspofungin, two with micafungin, and five with anidulafungin [20,26]. However, it has to be stated that the frequency of the paradoxical effect reported for the individual echinocandins but also for isolates of different fungal species differs greatly between different studies (Table 1). For example, paradoxical growth of *C. albicans* in the presence of caspofungin was observed by Chamilos et al. in 12 of 20 clinical isolates (60%) and by Fleischhacker et al. in only 14 of 101 clinical isolates (14%) [21,22]. A recently published study systematically assessed the occurrence of the paradoxical effect for a large number of *Candida* isolates (*n* = 602) treated with caspofungin, anidulafungin, and micafungin [24]. Again, the results regarding the frequency and drug dependency differed in some cases for the individual species when compared to previous studies (Table 1). The authors of this study proposed that the discrepancies in results could be related to methodological differences between the studies such as the chosen definition of paradoxical growth or the in vitro testing protocols, which were either EUCAST (European Committee on Antimicrobial Susceptibility Testing)- or CLSI (Clinical and Laboratory Standards Institute)-based and apply different media [24].

## 3. Factors That Influence the Manifestation of the Paradoxical Effect

Apart from the chosen echinocandin and the examined species and strain, several additional external factors influence the manifestation of the paradoxical effect. These include the media composition, media additives but also the exact culturing conditions such as steadiness of the echinocandin concentration, the presence of additive chemicals or physical stress, or growth in a planktonic versus sessile phase. For example, Stevens et al. have shown that paradoxical growth of *C. albicans* is more pronounced in synthetic amino acid medium-fungal (SAAMF) compared to RPMI 1640 medium, and less pronounced in yeast nitrogen base medium (YNB). Since RPMI 1640 and SAAMF are both buffered to physiologic conditions, the differences do not solely depend on the pH [18]. Supplementation of RPMI 1640 with a high concentration of serum (50%) was shown to abolish the paradoxical effect of caspofungin. Interestingly, a lower serum concentration (10%) did not abolish but rather drastically increased the concentration required to induce the paradoxical effect [23]. At the same time, the MIC of caspofungin was not (10%) or only moderately (50%) affected by serum [18,23]. Lewis and colleagues reported that supplementation of RPMI with 5% mouse serum can suppress the paradoxical effect of caspofungin on *A. fumigatus*. However, these results should be considered with some caution because they are solely based on measuring the metabolic activity of *Aspergillus* mycelium [27]. In another study, 50% serum was able to suppress paradoxical growth of *A. fumigatus* and other *Aspergillus* species [28]. Notably, the effect of serum on the MIC or MEC (in the case of *Aspergillus* spp.) of echinocandins is controversial and was summarized and discussed elsewhere [29].

Another factor that was repeatedly reported to affect the occurrence of a paradoxical effect is sessile growth within a biofilm as compared to conventional growth as planktonic cells [25,30,31]. Melo et al. analyzed the manifestation of the paradoxical effect for several isolates of *C. albicans*, *C. tropicalis*, *C. parapsilosis*, *C. orthopsilosis*, and *C. metapsilosis*. Interestingly, paradoxical growth was more frequently observed when the isolates were grown as biofilms (24 of 30 isolates; 80%) compared to planktonic cells culturing conditions (12 of 30 isolates; 40%). Besides this, individual isolates also had a different concentration range for the occurrence of the paradoxical effect when grown as a biofilm [30]. These results were confirmed by Ferreira et al. [31]. Later, Walraven et al. performed similar experiments with clinical *Candida* isolates that harbor defined echinocandin resistance mutations in the *fks1* gene, which encodes the β-GS. As expected, the echinocandin MICs of the different isolates were significantly increased when compared to susceptible strains. At the same time, the majority of the isolates still demonstrated paradoxical growth in the presence of extremely high echinocandin concentrations. Again, manifestation of the paradoxical effect of individual isolates depended significantly on the culturing condition (biofilm vs. planktonic) but also on the applied echinocandin derivative [25].

An interesting factor that was reported to significantly influence the appearance of the paradoxical effect is the steadiness of the echinocandin concentration. Stevens et al. already observed that inconstant drug concentration, which may occur when testing the susceptibility with a disc diffusion-like method on solid medium (agar), seems to suppress the paradoxical effect [18]. Similar observations were made by Shields et al. when investigating the postantifungal effect of caspofungin. They found that removal of caspofungin after exposure in a time-kill assay abolishes paradoxical growth. Interestingly, the intermittent exposure not only suppressed paradoxical growth but additionally increased the fungicidal activity of caspofungin when compared to unaltered or renewed drug exposure [32,33].

One important factor that determines the manifestation of the paradoxical effect is the echinocandin concentration itself. Apparently, the effect is only manifest in a certain high concentration range (approximately 4 to 32 µg mL^−1^) [17,18,19]. The reason for the absence of paradoxical growth at concentrations below this range but above the MIC or MEC (approximately 0.03 to 1 µg mL^−1^) is not known. However, we recently answered the question of why the paradoxical effect seems to disappear at very high concentrations (approximately >64 µg mL^−1^). We could show that the echinocandin caspofungin exerts additional antifungal activity against *A. fumigatus* besides inhibition of the β-GS at very high concentrations [34]. This additional activity suppresses the growth of otherwise paradoxically growing colonies and thereby explains the visual disappearance of the effect [34]. The exact mechanism behind the additional antifungal activity, however, remains unknown. Although possible alternative modes of action of echinocandins were proposed previously—for example, inhibition of β-1,6-glucan synthesis or induction of apoptosis [35,36,37]—none has been further elaborated or validated in successive studies.

## 4. The Clinical Relevance of the Paradoxical Effect

The question of whether the paradoxical effect is a mere in vitro phenomenon or could be of relevance in a clinical setting for the treatment of fungal infections was repeatedly raised and debated [18,33,38,39,40]. Unfortunately, this question still lacks a conclusive answer. So far, no clinical data directly support the hypothesis that the paradoxical effect could interfere with antifungal chemotherapy of candidiasis in humans [41,42,43,44,45]. Similar, the results of experimental studies in animal models that explored the potential benefit of high-dose echinocandin therapy against *Candida* species did not confirm the clinical relevance of the phenomenon [46,47]. On the contrary, several other experimental studies in murine or rabbit invasive aspergillosis models that focused on the efficacy of echinocandin dose escalation suggested a possible role of the paradoxical effect, mostly reflected by a moderate increase in the fungal burden at higher doses [27,48,49,50]. A similar result was obtained in a murine intraperitoneal candidiasis model in a study that specifically addressed the role of paradoxical growth of a *C. tropicalis* isolate treated with caspofungin [51]. Interestingly, one study that investigated the paradoxical effect in a neutropenic murine model of invasive aspergillosis specifically associated the phenomenon with caspofungin and not with micafungin, which would be in good agreement with the differing in vitro data for these two echinocandins [20,27]. Nevertheless, the repeatedly described paradoxical effect in animal models finally appeared to not significantly affect the overall survival [27,48,49,50,51].

Taken together, the evidence obtained with animal experiments suggests the occurrence of the paradoxical effect under in vivo conditions. However, the clinical significance remains unknown. It has to be noted that several of the paradoxical effect influencing the conditions mentioned above specifically play a role under in vivo conditions. Serum or similar factors could possibly suppress the manifestation of the effect. Continuous undulation of blood echinocandin levels during therapy as well as the chosen echinocandin derivative (caspofungin vs. micafungin/anidulafungin) could hinder manifestation as well. On the other hand, factors such as growth as a sessile pathogen in a microbial biofilm, a condition typically associated with catheter or implant infections, could facilitate a paradoxical effect that subsequently promotes the persistence of the infection in a niche under high-dose echinocandin therapy. Furthermore, clinically measurable significance might also be restricted to certain fungal species. For example, while *C. dubliniensis* showed paradoxical growth in up to 90% of the tested isolates [22], *C. glabrata* isolates almost never show the phenomenon [21,23,24]. Finally, the introduction of new echinocandin derivatives with exceptionally long-lived pharmacokinetic profiles, such as the echinocandin CD101 [52,53,54,55], could open up the question of whether the paradoxical effect manifests in vivo under constantly high drug levels.

## 5. Mechanistic Insights and Possible Signaling Pathways Involved

Despite great efforts to determine the factors that promote or repress paradoxical growth, the genetic and mechanistic basis of this phenomenon remains largely elusive. Many initial hypotheses to explain the paradoxical effect could be excluded: It has been demonstrated that paradoxical growth is not a result of resistance-associated mutations in the β-GS, upregulation of β-GS activity, inactivation, precipitation or degradation of the drug, or selection of a resistant subpopulation [18,19,56].

It was repeatedly reported that exposure to inhibitory echinocandin concentrations triggers a massive increase of cell wall chitin in *Candida* as well as in *Aspergillus* [14,34,56,57,58,59,60]. A similar drastic increase in cell wall chitin was observed after inhibition of β-GS expression in *A. fumigatus* [14]. This demonstrated that fungi must have a conserved stress response pathway that induces chitin synthesis upon depletion of cell wall glucan.

In fact, up to three signaling pathways were identified to upregulate chitin synthesis in response to echinocandin treatment. First, Walker et al. found that in *C. albicans* the cell wall integrity (CWI), the high osmolarity glycerol (HOG), and the calcineurin signaling pathways mutually stimulate the chitin synthases Chs2 and Chs8 to overcome echinocandin-induced stress [59]. Similar results were obtained for *A. fumigatus*. We have shown that disruption of the Wsc1 signaling branch of the CWI pathway increases the susceptibility of *A. fumigatus* to echinocandins [61]. However, direct evidence that links the susceptibility of such CWI signaling mutants with chitin synthesis is still lacking. The increase in total chitin synthase activity in the presence of caspofungin has been shown to be calcineurin-dependent [57]. This was revealed by analyses of *A. fumigatus* deletion mutants of the calcineurin A catalytic subunit CnaA and the downstream calcineurin-responsive zinc finger transcription factor CrzA. Interestingly, *cnaA* and *crzA* deletion mutants showed increased caspofungin susceptibility and no paradoxical growth [57]. Later, Lamoth et al. demonstrated that heat shock protein 90 (Hsp90) is involved in *A. fumigatus* calcineurin-dependent stress response to echinocandins. Similar to the *cnaA* and *crzA* mutants, inhibition of Hsp90 function results in increased caspofungin susceptibility and abolishes paradoxical growth [62,63].

**Figure 1:**
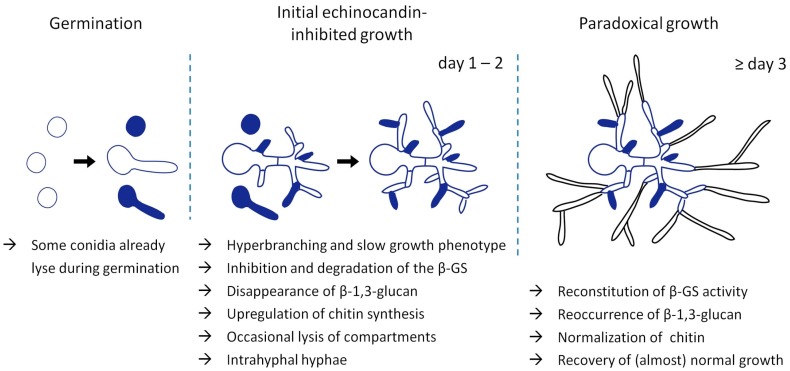
Manifestation of paradoxical growth of *A. fumigatus* exposed to echinocandins. A limited number of conidia do not survive germination in the presence of echinocandin antifungals. Surviving microcolonies exposed to high echinocandin concentrations show a slow growth phenotype characterized by hyperbranching, occasional lysis of hyphal compartments (colored compartments), translocation of the β-GS to vacuoles, disappearance of cell wall β-1,3-glucan and compensatory increase of cell wall chitin (blue cell walls). Regenerative intrahyphal growth initiated from the septa occurs in some lysed compartments. After approximately 2–3 days, paradoxically growing hyphae emerge from the β-1,3-glucan-depleted and growth-inhibited microcolonies. These paradoxically growing hyphae are characterized by fast growth, normal morphology, renewed localization of the β-GS to the hyphal tips, reconstitution of β-1,3-glucan synthesis, and normalization of the cell wall chitin levels.

Based on these results, it was tempting to speculate that the echinocandin-induced increase in cell wall chitin mediates paradoxical growth. However, we have recently shown that the chitin levels of paradoxically growing *Aspergillus* hyphae return to normal when compared to echinocandin-inhibited but non-paradoxically growing hyphae [34]. Moreover, we found that the paradoxical effect depends on expression of the β-GS and that paradoxically growing hyphae expose β-1,3-glucan again [34]. Importantly, the paradoxically growing hyphae show a rather normal morphology and emerge from an initially growth-inhibited, β-1,3-glucan-depleted, chitin-rich and hyperbranched mycelium that shows frequent hyphal lysis phenomena [14,34] (Figure 1). Interestingly, it takes approximately two to three days before any paradoxical growth can be observed [34]. These results were also confirmed by Moreno-Velásquez et al. [64]. Besides the switch in morphology, they showed that exposure to growth-inhibitory echinocandin concentrations causes a mislocalization of the β-GS to vacuoles. As soon as paradoxical growth begins, the β-GS showed renewed localization at the hyphal tips [64]. In summary, these data demonstrate that the paradoxical effect of echinocandins in the first line relies on the reconstitution of the β-GS activity.

Nevertheless, the initial increase in cell wall chitin that is regularly observed in echinocandin-inhibited non-paradoxically growing fungi could represent an essential precondition. It has been shown for multiple fungal species that inhibition of chitin synthesis strengthens the fungicidal activity of echinocandins [23,65,66]. Consequently, the initial increase in cell wall chitin facilitates survival of the organisms upon inhibition of β-1,3-glucan synthesis. Survival is, of course, a prerequisite for the subsequent reconstitution of the β-GS activity and manifestation of the paradoxical effect (Figure 1). Still, the mechanism by which the fungus reconstitutes β-1,3-glucan synthesis remains unresolved.

The interesting observation of Moreno-Velásquez et al. that the β-GS localizes to vacuoles under growth inhibitory caspofungin concentrations suggests that echinocandins could change the enzyme’s conformation, thereby committing inhibited β-GS to degradation. Renewed localization to the hyphal tips and reconstitution of β-1,3-glucan synthesis during paradoxical growth could then indicate a conserved mechanism to stabilize or protect newly synthesized β-GS. It is, however, surprising that the manifestation of such a salvage mechanism appears to significantly depend on the echinocandin derivative, occurs only in certain strains and species, and requires days to manifest. To this end, many open questions remain about this mysterious echinocandin-specific phenomenon and should be addressed in future studies.

## Figures and Tables

**Table 1:** Manifestation frequency of the paradoxical effect in vitro in percent (%) of caspofungin (CS), micafungin (MI), and anidulafungin (AN) among clinical isolates of selected *Candida* and *Aspergillus* species in different studies. The numbers of tested isolates (n) are given in brackets. Paradoxical growth was determined with CLSI (Clinical and Laboratory Standards Institute)-[21,22,23], EUCAST (European Committee on Antimicrobial Susceptibility Testing)-[24], or metabolism-based [20] broth microdilution methods in RPMI 1640-based media.

Study	*C. albicans* (CS/MI/AN)	*C. glabrata* (CS/MI/AN)	*C. tropicalis* (CS/MI/AN)	*C. dubliniensis* (CS/MI/AN)	*C. parapsilosis* (CS/MI/AN)	*A. fumigatus* (CS/MI/AN)	*A. terreus* (CS/MI/AN)	*A. flavus* (CS/MI/AN)
Chamilos et al. [21]	60/0/40 (*n* = 20)	0/0/0 (*n* = 10)	40/70/20 (*n* = 10)		90/0/0 (*n* = 10)			
Antachopoulos et al. [20]						55/18/45 (*n* = 11)	63/13/25 (*n* = 8)	13/0/0 (*n* = 8)
Fleischhacker et al. [22]	14/0/0 (*n* = 72–103)			90/63/0 (*n* = 124–127)				
Shields et al. [23]	60/13/23 (*n* = 30)	0/0/0 (*n* = 34)			4/0/0 (*n* = 23)			
Marcos-Zambrano et al. [24]	10/<1/11 (*n* = 291)	0/2/3 (*n* = 62)	62/38/48 (*n* = 50)		6/1/1 (*n* = 164)			
